# Assessment of *TP53* Mutations in Benign and Malignant Salivary Gland Neoplasms

**DOI:** 10.1371/journal.pone.0041261

**Published:** 2012-07-19

**Authors:** Carolina Cavaliéri Gomes, Marina Gonçalves Diniz, Lissur Azevedo Orsine, Alessandra Pires Duarte, Thiago Fonseca-Silva, Brendan I. Conn, Luiz De Marco, Cláudia Maria Pereira, Ricardo Santiago Gomez

**Affiliations:** 1 Department of Pathology, Biological Sciences Institute, Universidade Federal de Minas Gerais, Belo Horizonte, Brazil; 2 Department of Oral Surgery and Pathology, School of Dentistry, Universidade Federal de Minas Gerais, Belo Horizonte, Brazil; 3 Department of Pathology, Royal Infirmary of Edinburgh, Edinburgh, Scotland; 4 Department of Surgery, School of Medicine, Universidade Federal de Minas Gerais, Belo Horizonte, Brazil; Harvard Medical School, United States of America

## Abstract

Despite advances in the understanding of the pathogenesis of salivary gland neoplasms (SGN), the molecular pathways associated with enhanced tumor growth and cell survival remain to be established. The aim of the present study was to investigate whether *TP53* mutations are relevant to SGN pathogenesis and if they impact on p53 protein expression. The study included 18 benign and 18 malignant SGN samples. Two polymorphic microsatellite markers at the *TP53* genetic locus were chosen to assess loss of heterozygosity (LOH) in the samples that had matched normal DNA. The *TP53* exons 2–11 were amplified by PCR, and all of the products were sequenced. Reverse transcription-PCR of the *TP53* open reading frame (ORF) was carried out in the samples that had fresh tissue available, and immunohistochemistry for the p53 protein was performed in all samples. *TP53* LOH was only found in two pleomorphic adenomas. We found two missense mutations in exon 7 (one in a pleomorphic adenoma and the other in a polymorphous low grade adenocarcinoma), another in exon 8 (in a carcinoma ex pleomorphic adenoma) and a fourth missense mutation in exon 10 (in a mucoepidermoid carcinoma). In addition, a nonsense mutation was found in exon 8 of an adenoid cystic carcinoma. Several intronic and exonic SNPs were detected. Although almost all of the malignant samples were immunopositive for p53, approximately 37% of the benign samples were positive, including the sample harboring the missense mutation and one of the samples that showed LOH. The complete *TP53* ORF could be amplified in all samples analyzed, including the IHC negative samples, the samples showing LOH and one sample displaying a missense mutation. In summary, our results show that *TP53* mutations are not a frequent event in SGN and that p53 immunopositivity might not be associated with sequence mutations in SGN.

## Introduction

Salivary gland tumors are uncommon neoplasms that primarily affect the major salivary glands. Salivary gland neoplasms (SGN) have an annual global incidence of 0.4 to 13.5 cases per 100,000 individuals [1]. There are no data regarding the incidence of SGN in Brazil, yet the prevalence of each group of tumors is similar to that reported globally [2], [3]. Current treatment relies on surgical excision and, if appropriate, postoperative radiotherapy. Previous work indicates that tumors smaller than 4 cm (T1 or T2) do well, regardless of histological type or grade [4]. Unresectable or recurrent tumors may respond to chemotherapy, although the results are very modest [5], [6].

*TP53* is located at 17p 13.1, and it is altered in many types of cancer. It has been shown to contain one of the initiating mutations in the majority of ulcerative colitis-associated neoplasias [7]; however, the role of p53 in salivary gland neoplasms is controversial and needs to be clarified [1].

There is little information about *TP53* alterations in salivary gland tumors. Previous papers reported positive immunoexpression of p53 protein [8], [9], yet *TP53* mutations seem to be infrequent events in pleomorphic adenomas (PA), adenoid cystic carcinomas (ACC), mucoepidermoid carcinomas (MEC) and carcinomas ex pleomorphic adenomas (CAexPA) [10], [11], [12], [13], [14]. These previous papers relied on different techniques (SSCP, for example, which misses mutations when compared to direct sequencing), and in some of them, sequencing was only performed on a few samples. In salivary gland neoplasms, LOH at the *TP53* locus has also been reported, mainly in malignant salivary neoplasms or based on visual comparison of the intensity of the alleles [15], [16], [17], [18]. It is important to note that some *TP53* mutations do not result in positive immunostaining. However, p53 protein accumulation can occur in the absence of underlying gene mutations [19] in response to cellular stress that can result in stabilization, accumulation and activation of p53 in the nucleus [20].

Despite the low reported rates of *TP53* mutations in SGN, the screening of more samples by direct sequencing is necessary in the era of personalized medicine that includes selecting chemotherapeutic agents based on the identification of particular molecular lesions in a tumor. For example, some *TP53* mutations confer sensitivity to cisplatin-induced apoptosis [21]. In this sense, it is mandatory to clarify the role of *TP53* alterations in salivary tumors. It would be interesting, for example, to find which specific tumors harbor specific *TP53* mutations because individual patients might benefit from specific drugs. There is little information on this subject in the literature, and it has been reported that unresectable or recurrent salivary tumors may respond to chemotherapy [5], [6]. Why do some patients fail to benefit from chemotherapy? A better molecular characterization of tumors can be translated to more accurate treatments.

On the basis of this evidence, we screened all *TP53* coding exons for mutations and assessed LOH at the *TP53* locus in a subset of benign and malignant salivary gland neoplasms and performed p53 immunohistochemistry on the samples. The aim of the present study is to assess whether *TP53* mutations are relevant to SGN pathogenesis and if they impact on p53 expression.

## Materials and Methods

### Ethics Statement

The local Ethics Committee (Universidade Federal de Minas Gerais) approved the work, and signed written consent was obtained from each patient.

### Tissue and DNA Extraction

A total of 36 salivary gland neoplasms were included in the study, 18 benign (Table 1) and 18 malignant (Table 2). Briefly, a portion of each lesion was removed on resection, immediately snap frozen in liquid nitrogen and stored at −80°C. To confirm the original diagnosis, an adjacent sample was fixed in 10% buffered formalin and embedded in paraffin. Routine hematoxylin- and eosin-stained sections were reviewed by three independent pathologists. Samples of the solid tumors were dissected using frozen section controls. DNA was extracted from fresh tissue with QIAamp DNA Mini kits (Qiagen, Hilden, Germany), according to the manufacturer’s instructions. The DNA of nine samples (one PLGA  =  polymorphous low grade adenocarcinoma, two MEC, two ACC and four CAexPA) was obtained from formalin fixed, paraffin embedded (FFPE) material. The CAexPA samples consisted of three invasive tumors (samples #31, #33 and #34) and one non-invasive (sample #32). The DNA obtained from these microdissected solid tumor samples was extracted with QIAamp DNA FFPE Tissue Kits (Qiagen, Hilden, Germany). The carcinoma component of the CAexPA samples was microdissected prior to DNA extraction. Peripheral blood from 16 of these patients and normal oral mucosa from one patient were used as matched normal DNA controls (Tables 1 and 2).

**Table 1:** Clinical data, immunohistochemistry (IHC), LOH and *TP53* mutations summarized results of benign salivary gland neoplasms.

Sample	Age	Location	T size	IHC	17p markers		Exons										Introns				
					TP53	AFM238 WF2	e2	e3	e4	e5	e6	e7	e8	e9	e10	e11	i2	i7	i8	i9	i10
Pleomorphic Adenoma (n = 16)																					
**1**	67	hard palate	T2	+	*	*	no	no	S	no	no	no	no	no	no	no	S	S	no	no	no
**2**	28	soft palate	T2	+	○	○	no	no	S	no	no	no	no	no	no	no	S	S	no	no	no
**3**	44	upper lip	T1	+	*	*	no	no	S	no	no	no	no	no	no	no	S	S	no	no	no
**4**	16	hard palate	NA	**	*	*	no	no	S	no	no	no	no	no	no	no	S	S	no	no	no
**5**	25	parotid	T1	−	○	○	no	no	S	no	no	no	S	no	no	no	S	S	no	no	no
**6**	46	parotid	NA	−	□	○	no	no	S	no	no	no	no	no	no	no	S	S	S	no	no
**7**	51	parotid	NA	−	*	*	no	no	S	no	no	no	no	no	no	no	S	S	no	no	no
**8**	76	submandibular	T1	−	○	○	no	no	S	no	no	no	S	no	no	no	S	S	S	no	S
**9**	35	submandibular	T3	−	□	○	no	no	S	no	no	no	S	no	no	no	S	S	no	no	no
**10**	30	upper lip	NA	**	*	*	no	no	S	no	no	no	no	no	no	no	S	S	no	no	no
**11**	48	buccal mucosa	T2	+	*	*	no	no	S	no	no	M	no	no	no	no	S	S	no	no	no
**12**	73	soft/hard palate	T3	−	○	○	no	no	S	no	no	no	no	no	no	no	S	S	no	no	no
**13**	58	parotid	NA	+	*	*	no	no	S	no	S	no	no	no	no	no	S	S	no	no	no
**14**	22	parotid	T1	−	*	*	no	no	S	no	no	no	no	no	no	no	S	S	no	no	no
**15**	46	parotid	T1	−		○	no	no	S	no	no	no	no	no	no	no	S	S	no	no	no
**16**	20	buccal mucosa	T1	+		○	no	no	no	no	no	no	no	no	no	no	S	S	no	no	no
Basal Cell Adenoma (n = 1)																					
**17**	48	parotid	NA	−	○	□	no	no	S	no	no	no	S	no	no	no	S	S	no	no	no
Mucinous Cystadenoma (n = 1)																					
**18**	40	palate	NA	−	□	○	no	no	S	no	no	no	no	no	no	no	S	S	no	no	no

M = missense; S =  silent or SNP; * LOH analysis was not carried out in these samples, as they did not harbor normal tissue control or due to small amount of available tissue; ** the immunostaining was not done in these samples due to small amount of available tissue; NA =  not available; □ Homozygous ○ Heterozygous LOH (loss of heterozygosity); Not all introns were analyzed, as our primers were designed to sequence the exons.

**Table 2:** Clinical data, immunohistochemistry (IHC), LOH and *TP53* mutations summarized results of malignant salivary gland neoplasms.

Sample	Age	Location	T size	IHC	17p markers		Exons										Introns				
					TP53	AFM238 WF2	e2	e3	e4	e5	e6	e7	e8	e9	e10	e11	i2	i7	i8	i9	i10
Polymorphous Low Grade Adenocarcinoma (n = 4)																					
19	61	Soft palate	T3	+	*	*	no	no	S	no	no	no	S	no	no	no	S	S	S	no	no
20	64	Hard palate	NA	+	○	○	no	no	S	no	no	**M**	no	no	no	no	S	S	no	no	no
21	50	Soft palate	T2	+	○	○	no	no	S	no	no	no	no	no	no	no	S	S	no	no	no
22	67	Hard palate	T2	+	□	○	no	no	S	no	no	no	no	no	no	no	S	S	no	no	no
Mucoepidermoid Carcinoma (n = 4)																					
23	28	Hard palate	NA	+	*	*	no	no	S	no	no	no	no	no	no	no	S	S	S	no	no
24	53	NA	NA	+	*	*	no	no	S	no	no	no	no	no	**M**	no	S	S	no	no	no
25	25	Hard palate	T1	+	*	*	no	no	S	no	no	no	no	no	no	no	S	S	no	no	no
26	67	Parotid	NA	−	○	○	no	no	S	no	no	no	no	no	no	no	S	S	no	no	no
Adenoid Cystic Carcinoma (n = 4)																					
27	50	Hard palate	T4	+	*	*	no	no	S	no	no	no	no	no	no	no	S	S	S	no	no
28	66	Submandibular	NA	+	○	○	no	no	S	no	no	no	no	no	no	no	S	S	no	no	no
29	72	Parotid	T2	+	*	*	no	no	S	no	no	no	**N**	no	no	no	S	S	no	no	no
30	37	Parotid	T2	+	*	*	no	no	S	no	no	no	no	no	no	no	S	S	no	no	no
Carcinoma ex-Pleomorphic Adenoma (n = 4)																					
31	57	Parotid	T2	+	*	*	no	no	S	no	no	no	no	no	no	no	S	S	no	no	no
32	52	Parotid	T2	+	*	*	no	no	S	no	no	no	no	no	no	no	S	S	no	no	no
33	84	Parotid	T2	+	*	*	no	no	no	no	no	no	no	no	no	no	no	S	no	no	no
34	71	Parotid	T3	+	*	*	no	no	S	no	no	no	**M**,S	no	no	no	S	S	S	no	no
Basal Cell Adenocarcinoma (n = 1)																					
35	77	Buccal mucosa	T2	+	○	□	no	no	no	no	no	no	no	no	no	no	no	S	no	no	no
Cystadenocarcinoma (n = 1)																					
36	59	Submandibular	T3	−	○	○	no	no	no	no	no	no	no	no	no	no	S	S	no	S	no

M = missense; N =  nonsense; S =  silent or SNP;

* LOH analysis was not carried out in these samples, as they did not harbor normal tissue control; NA =  not available;

□Homozygous ○ Heterozygous LOH (loss of heterozygosity); Sample #19 disease recurred 6 years after diagnosis and patient #27 died of disease 4 years after diagnosis. DNA from samples #20,23,24,29–34 were retrieved from FFPE material. Not all introns were analyzed, as our primers were designed to sequence the exons.

### LOH at 17p 13.1

Two polymorphic microsatellite markers at the *TP53* genetic locus (17p 13.1) were selected for PCR (Table 3). Fluorescently labeled primers were used to yield PCR products of approximately 150 base pairs. Because LOH was determined by comparing the allele ratio in tumor DNA to the allele ratio in normal DNA [22], only tumors from patients from whom we had DNA from normal tissue were included in the LOH assay (i.e., 17 cases). PCR was performed with tumor and normal DNA under the same conditions using a 15 µl reaction mixture containing 0.25 µl (20 pmol/µl) of each primer, 150 ng of extracted DNA, 5 µl of deoxynucleoside triphosphate (0.25 µM of each triphosphate), 0.75 µl (1.5 mM) magnesium chloride, 2.5 µl (1X) of commercial PCR buffer, and 0.2 µl (1 unit) of Platinum Taq polymerase (Invitrogen, Carlsbad, CA, USA). The amplified PCR products were confirmed in an 8% polyacrylamide gel and run in an ABI PRISM 310 Genetic Analyzer (Applied Biosystems, Foster City, CA, USA), and the data were analyzed using GeneMapper software version 3.0 (Applied Biosystems, Foster City, CA, USA). LOH was calculated as the ratio between the short allele-normal (Sn)/long allele-normal (Ln) and short allele-tumor (St)/long allele-tumor (Lt) using the following formula: (Sn:Ln)/(St:Lt). LOH was scored when one allele (peak) was decreased by more than 50% in the tumor sample compared to the same allele in normal tissue, followed by DNA stutter correction when necessary (score <0.5 or >2) [22]. A sample was considered non-informative (NI) when the control DNA for normal tissue was homozygous for the polymorphic markers (i.e., showing only one peak in the normal control tissue).

**Table 3:** Markers at the 17p 13.1 locus used in the LOH analysis.

Biomarker	Primer sequences	Type of repetition	PCR product size
AFM238WF2^a^	F: AACAGCCTGTGCAACATAGT	Di (CA)	160 bp
	R: AGCTCGAAGCAACAACACTT		
*TP53* b	F: TACAGGGATAGGTAGCCCGAG	Di (CA)	149 bp
	R: GGATTTGGGCTCTTTTGTAA		

a GenBank Z66843.1.

b GenBank AB134622.1.

### *TP53* Mutations Analyzed by Direct Sequencing

Because *TP53* mutations occur throughout the gene in human cancer, we screened all of the *TP53* coding exons (2–11) for mutations by direct DNA sequencing. DNA fragments were amplified by PCR using primer pairs previously described (http://www-p53.iarc.fr) [23], with the exception of the primers used to sequence exons 2 and 3 (Table S1). GenBank accession number NC_000017.9 was used where the nucleotide +1 corresponds to the first A of exon 1. Amplified products were confirmed by electrophoresis on 6.5% polyacrylamide gels with silver staining. PCR products were purified with GFX PCR DNA and Gel Band Purification Kits (Amersham Biosciences, Piscataway, NJ, USA) and sequenced on an ABI PRISM 310 Genetic Analyzer (Applied Biosystems, Foster City, CA, USA).

### Immunohistochemistry

Briefly, 4 µm paraffin-embedded sections were dewaxed in xylene and hydrated with graded ethanol. Endogenous peroxidase was blocked by 1% hydrogen peroxide for 15 minutes. Antigen retrieval was performed in citric acid, pH 6.0. The samples were incubated with primary antiserum against p53 (clone DO7, Dako Cytomation, Glostrup, Denmark) diluted in BSA 0.5% at 1∶50 for 30 minutes at room temperature. Binding was visualized using a polymer-based system (EnVision, Dako Corporation, Carpinteria, CA, USA) with diaminobenzidine (Sigma, St Louis, MO, USA) as the chromogen. The epitope recognized by the antibody is possibly located between amino acids 37 and 45 of the human p53 protein. Positive controls (a squamous cell carcinoma with known reactivity) and negative controls (in which the primary antibody was omitted) were included. The sections were counterstained with hematoxylin, dehydrated and mounted. Nuclei with p53 staining were counted in eight fields (400x magnification). A sample with more than 5% positive nuclei was considered positive. Counting was performed by two pathologists independently.

### RNA Extraction and Reverse Transcription PCR (RT-PCR)

Total RNA from 27 fresh frozen samples was isolated with Tri-Phasis Reagent (BioAgency, São Paulo, Brazil). The cDNA was synthesized from 1.5 µg of DNAse-treated RNA by using Superscript First-Strand Synthesis System kits (Invitrogen Life Technologies Carlsbad, CA, USA). Two primers, F 5′-AGTCTAGAGCCACCGTCCA-3′ and R 5′-TCTGACGCACACCTATTGCAAGC-3′, were used to amplify the *TP53* open-reading-frame (ORF) with Platinum Taq DNA Polymerase (Invitrogen Life Technologies Carlsbad, CA, USA) as described elsewhere [24].

## Results

Table 1 shows a summary of the results of the benign samples and Table 2 summarizes the results of the malignant ones. The complete description of all results can be found in Table S2.

### Direct Sequencing Results

Exons 2, 3, 5, 9 and 11 did not show any sequence alterations. The alterations found in the other exons and in the introns are described below.

#### Missense and nonsense mutations

**Figure 1:**
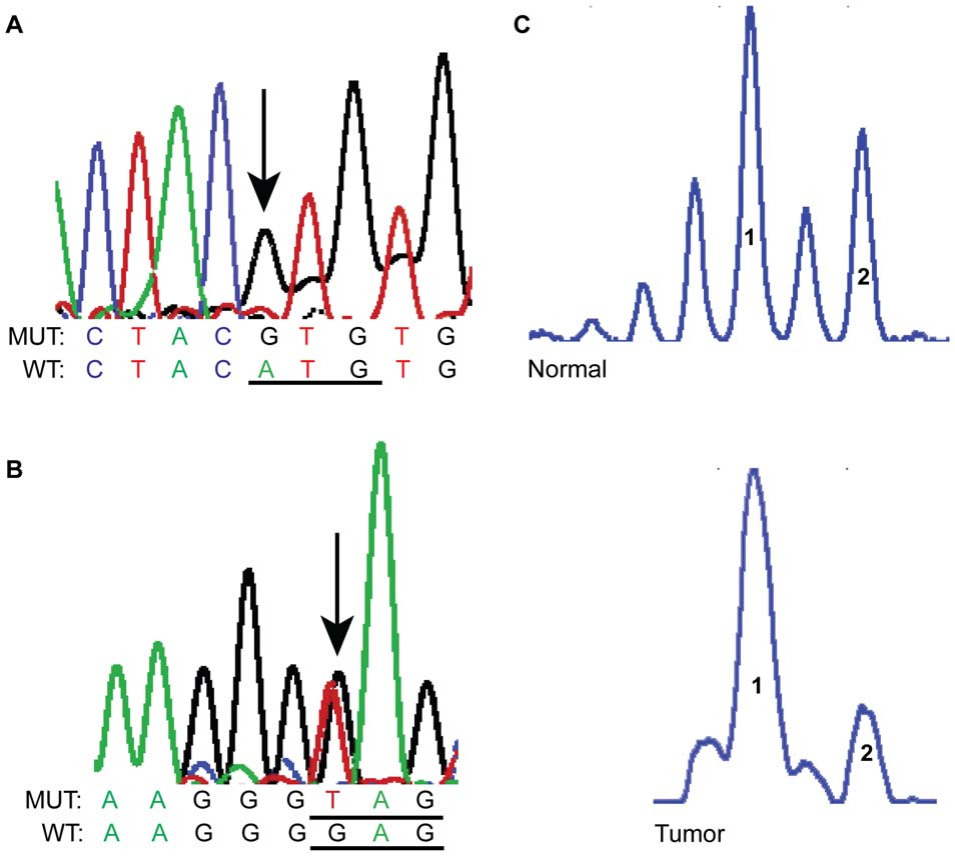
Representative SGN samples showing *TP53* missense mutations (A and B) and LOH (C) using the microsatellite marker TP53 located on chromosome 17 p. A and B are screen shots from sequencing electropherograms. **A**: A PLGA (sample # 20) showed a homozygous missense mutation (arrow) in exon 7 g.13346A>G (WT codon ATG→Mut codon GTG); **B**: ACC (sample #29) exhibiting a heterozygous nonsense mutation in exon 8, g.13860G>T (WT codon GAG→Mut codon TAG); **C**: Screen shots of electropherograms generated by GeneMapper. When comparing tumor sample with normal constitutive DNA, there was loss of the long allele. 1: short allele and 2: long allele. WT: wild type, MUT: mutant.

**Figure 2:**
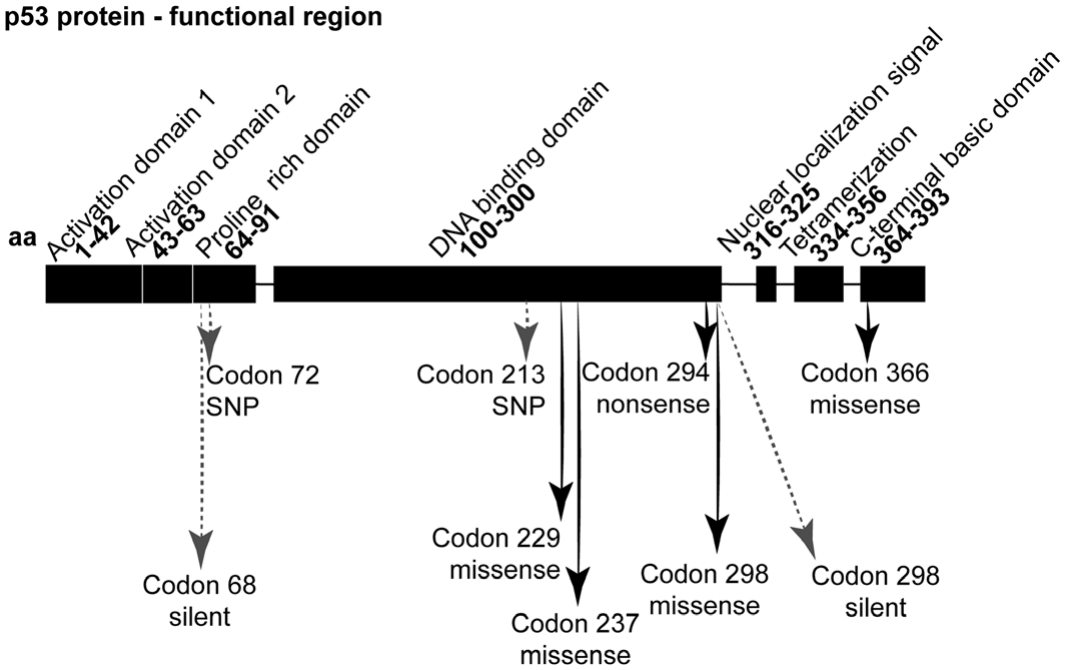
Localization of *TP53* mutations in relation to the p53 protein functional region. Note that 4/5 missense and nonsense mutations occurred in the DNA binding domain. Black arrows  =  missense and nonsense mutations, grey arrows  =  SNPs and silent mutations.

We found four missense mutations and one nonsense mutation (Table 4). Four of these five mutations were found in malignant samples (4/18), while the other one was found in a benign tumor (1/18). Three of these five tumors for which the tumor size was available were T2 or T3. The screen shots from sequencing electropherograms of two of these missense mutations is shown in Figure 1, and the localization in relation to the p53 protein functional region is illustrated in Figure 2.

**Table 4:** Missense and nonsense *TP53* mutations.

#	Exon	Sample	Codon #	WT→Mutantcodon	Effect
1	Exon 7	PA (#11)	Codon 229	TGT→AGT	Missense
2		PLGA (#20)	Codon 237	ATG→GTG	Missense
3	Exon 8	CaexPA (#34)	Codon 298	GAG→CAG	Missense
4		ACC (#29)	Codon 294	GAG→TAG	Nonsense
5	Exon 10	MEC (#24)	Codon 366	TCC→ACC	Missense

Mutations #1–4 have been previously reported as somatic mutations in other tumour types at the IARC *TP53* database. Mutation #2 was not carried by the patient blood, meaning it was a somatic mutation. The others could not be evaluated in blood, as it was not available for analysis. PA =  pleomorphic adenoma; PLGA =  polymorphous low grade adenocarcinoma; CaexPA =  Carcinoma ex-pleomorphic adenoma; ACC =  adenoid cystic carcinoma; MEC =  mucoepidermoid carcinoma. WT =  wild-type.

#### Variations and silent mutations in exons

In exon 4, the missense SNP, g.11446C>G (rs1042522, codon 72), leading to a proline to arginine substitution (also known as R72P) was detected in almost all samples, either in the homozygous or heterozygous form. A silent substitution in exon 4, g.11435G>A (codon 68), was detected in one sample (#26) but was not detected in the patient’s blood, suggesting that it is a somatic mutation. The silent SNP, rs1800372 in exon 6 (codon 213), was also found in only one sample (#13).

The other substitution found, exon 8 g.13874G>A (codon 298), is silent and has been previously reported as a somatic mutation in one breast cancer and one skin cancer sample in the IARC p53 database (http://www-p53.iarc.fr/, version R15, November 2010). However, this substitution was found in five of our samples and confirmed in the blood of 4/4 patients that had matched blood samples, suggesting that it is a genetic polymorphism rather than a somatic mutation.

The silent substitution in codons 68 and 298 and rs1800372 have been analyzed at the IARC p53 database with splicing prediction tools available on the web (NNSPLICE 0.9 and HSF V2.3). Apparently, none of them lead to significant changes on splicing. The localization of the variations and silent mutations in relation to the p53 protein functional region is illustrated in Figure 2.

#### Variations in introns

Intron 2 SNP g.11117C>G (rs1642785) was found at a very high frequency.

In intron 7, the previously described SNP rs67056327 was found in only one sample, but the SNP rs1642786 and the heterozygous substitution, g.13465G>A, were found in most of the samples. This intronic substitution was previously reported in one nasal cavity tumor sample (http://www-p53.iarc.fr/). We sequenced the DNA from the blood of some patients and found that the same mutation was carried by the patients’ germline DNA, excluding its somatic origin. We further analyzed DNA from normal oral mucosa of healthy individuals, and 7/8 individuals showed heterozygosity at this nucleotide, suggesting that it is a polymorphism. The SNP rs1642786 was also confirmed in these germline DNAs.

In intron 8, we found four heterozygous substitutions, including two (g.13911 G>A and g.13914C>T) previously reported (http://www-p53.iarc.fr/), and two (g.13916A>G and g.13941G>A) that had never been reported in the IARC p53 (version R15, November 2010), dbSNP (http://www.ncbi.nlm.nih.gov/snp/) or Ensembl (http://www.ensembl.org/index.html) databases. The g.13941G>A substitution was confirmed in matched blood DNA, proving that it is not somatic; however, the g.13911G>A, g.13914C>T and g.13916 A>G substitutions were only found in one sample each, and no blood or normal tissue was available to confirm them as polymorphisms.

Two other SNPs were found in one sample each: SNP rs1800899 in intron 9 and SNP rs17880847 in intron 10.

#### LOH results

The results of *TP53* sequencing and LOH are displayed in Tables 1 and 2. The LOH at the *TP53* locus was only found using the microsatellite markers at the *TP53* locus in two PAs. The frequency of allelic loss for the TP53 marker was 15.38% (two of 13 informative cases showed LOH). The heterozygous substitutions found during sequencing constitute additional evidence of the heterozygosity of *TP53* (i.e., absence of LOH) in our samples. Figure 1 shows an example of LOH at the marker TP53.

#### Immunohistochemistry results and RT-PCR results

**Figure 3:**
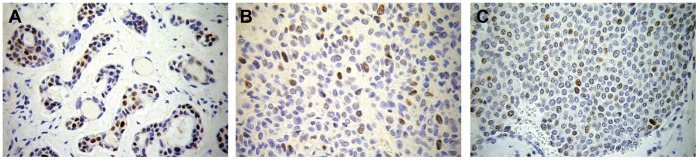
Immunohistochemistry photomicrographs showing p53 expression in three positive samples. A =  adenoid cystic carcinoma (original magnification 200x), B and C =  Carcinoma ex-Pleomorphic Adenoma (original magnification 400x).

The immunohistochemistry results are displayed in Tables 1 and 2. Although almost all malignant samples were positive for p53 staining, only approximately 37% of the benign samples were positive, including the sample harboring the missense mutation and one of the samples that showed LOH. Figure 3 shows the immunostaining pattern of p53.

The full length *TP53* ORF from 26 of the fresh frozen samples, including 16 PAs, one basal cell adenoma (BCA), one mucinous cystadenoma (MCA), three PLGAs, two ACCs, two MECs and one cystadenocarcinoma (CA), was amplified by RT-PCR. The complete *TP53* ORF could be amplified in all of the samples analyzed, including the IHC negative samples, the samples showing LOH and one sample displaying a missense mutation.

## Discussion

*TP53* mutations are found in a high percentage of human tumors and have been shown to be one of the initiating mutations in the majority of ulcerative colitis-associated neoplasias [7]. However, there is little information about SGN *TP53* mutations from direct sequencing of all samples [14].

The *TP53* gene is located at 17p 13.1. A comparative genomic hybridization (CGH) study of benign salivary gland tumors (15 Warthin tumors and 14 PA) found 17p 13.1 deletions in 11 of the 29 samples [25]. Fowler and co-workers found LOH at 17p 13 (p53) in 73% of the malignant mixed tumors that they analyzed [16]. In the present paper, LOH at 17p 13.1 using microsatellite markers was observed only in 2/13 informative samples. In addition, because we found heterozygosity of the *TP53* gene in several tumor samples by direct sequencing, we conclude that LOH of the gene is an unusual event in this subset of SGN.

While Augello and colleagues focused on exons 5–8 and found mutations in 3/28 PA and 2/4 ACC [10], Gedlicka *et al.* found no evidence of mutations in *TP53* exons 2–11 in 14 PA and 11 CAexPA [11]. Kiyoshima *et al.* found *TP53* mutations in 3/17 ACC ad 3/27 MEC when analyzing exons 5–8 [13]. Kishi *et al.* investigated *TP53* mutations in exons 5–8 of salivary carcinomas and only found mutations in 7 of 33 cases [12]. All these studies relied on either DGGE or SSCP assays, followed by direct sequencing when necessary. Other groups assessed *TP53* mutations by performing SSCP analysis alone and observing band shifts in exons 5, 6 and 8 in salivary tumors; however, they did not sequence the samples [26], [27]. SSCP can miss mutations detected by direct sequencing. Weber and co-workers analyzed *TP53* mutations by direct sequencing of exons 4–9 and detected mutations in 4/42 PA and 3/12 myoepitheliomas [14]. Despite the evidence that *TP53* might be altered in SGN [10], [13], [14], [16], [17], [18], we sequenced all of our samples and found only four missense mutations, one nonsense *TP53* mutation and a very low frequency of LOH at the 17p 13.1 region in our subset of salivary tumor samples.

In the present study, the samples that showed missense and nonsense mutations and one sample that showed *TP53* LOH were p53 positive by immunohistochemistry. There is increasing evidence that noncoding DNA changes may affect disease susceptibility [28], and *TP53* intronic mutations have been previously detected in a variety of tumors [29], [30]. Without a functional study, we cannot know if the intronic mutations we found had an effect on the p53 protein product. In some tumors, for instance, *TP53* intronic mutations have been associated with stabilization of the p53 protein [31]. On the other hand, p53 protein can accumulate in the absence of underlying gene mutations [19] in response to cellular stress that can result in stabilization, accumulation and activation of p53 in the nucleus [20]. The exact role of this p53 protein accumulation in tumors has not been completely clarified. In principle, p53 accumulation without *TP53* mutations could decrease the anti-apoptotic activity action that is favorable to the tumor. Although p53 accumulation is associated with a significantly lower apoptotic index in corticotroph adenomas [32], it might not be important for the apoptotic activity in thyroid neoplasms [33].

We directly sequenced the DNA binding site of the p53 protein (exons 5 to 9), as well as all the other coding exons of *TP53* (i.e., 2, 3, 4, 10, and 11) of all samples, and found a low coding change mutation rate (1/18 benign samples and 4/18 malignant samples). However, there is a possibility that some mutations may have been missed in our study because direct sequencing only detects mutations when a percentage of cells (approximately 20%) have the alteration. We did repeat some sequencing reactions with different DNA samples of the same cases, but the results were all similar.

Our results contribute to the ongoing debate on the role of *TP53* in the pathogenesis of salivary gland tumors. We found synonymous and intronic *TP53* mutations, but the *TP53* ORF was detected in all tested samples. The low frequency of coding change mutations and the low LOH rates suggest that alterations in this gene are most likely not early events in the development of salivary gland neoplasms. This possibility has implications for the molecular diagnoses that will be used to characterize the mutations shown to be important in personalized medicine. *TP53* mutations can confer sensitivity to cisplatin-induced apoptosis [21]. To date, small patient series, often including patients with a number of different histological subtypes of SGN, have been reported using several different, largely cisplatin-containing drug combinations with modest response rates [5]. In this sense, *TP53* mutation screening could be important to select the patients that would benefit from chemotherapy and those who would not. Furthermore, some *TP53* SNPs are known to modulate responses to chemotherapy [29]. The exon 4 SNP R72P (rs1042522) results in a structural change of the protein. In a cohort with head and neck cancer, the response to chemotherapy and survival rate were significantly higher in cases retaining the allele encoding arginine [34]. Although only five cases in our study had coding change mutations, some tumors did show silent and intronic mutations. Currently, the importance of such mutations is not clear because even silent mutations are not necessarily neutral [35]. Functional studies may prove that even intronic *TP53* mutations are important in personalized therapy. Finally, emerging high throughput technologies, such as exome sequencing, will be valuable for detecting disease-causing variants of *TP53* in salivary gland neoplasms just as in some other types of cancer, such as oral cancer [36], [37], [38].

### Conclusions

In conclusion, our results show that *TP53* mutations are not a frequent event in SGN and that p53 immunopositivity is not associated with sequence mutations.

## Supporting Information

Table S1 **Primers used in** ***TP53*** **sequencing. Primers previously described at IARC p53 database** [**23**] **(** http://www-p53.iarc.fr **).** *Designed using Primer Express software (Applied Biosystems, Foster City, CA, USA) version 3.0.
(DOC)

Table S2 **Expanded results of** ***TP53*** **mutations assessment, loss of heterozygosity and immunohistochemistry of all salivary gland neoplasms included in the study.** * LOH analysis was not carried out in these samples, as they did not harbor normal tissue control or due to small amount of available tissue; **the immunostaining was not done in these samples due to small amount of available tissue; NA =  not available; □ Homozygous ○ Heterozygous LOH (loss of heterozygosity); Sample #19 disease recurred 6 years after diagnosis and patient #27 died of disease 4 years after diagnosis. DNA from samples #20,23,24,29–34 was retrieved from FFPE material. Not all introns were possible to be analyzed, as our primers were designed to sequence the exons.
(XLS)
