# Supplementary material for: Assessment of TP53 Mutations in Benign and Malignant Salivary Gland Neoplasms
Source: PLoS One. 2012 Jul 19;7(7):e41261. doi: 10.1371/journal.pone.0041261 (PMC3400573; doi:10.1371/journal.pone.0041261)
Supplement: Table S1 — Primers used in TP53 sequencing. Primers previously described at IARC p53 database [23] ( http://www-p53.iarc.fr ). *Designed using Primer Express software (Applied Biosystems, Foster City, CA, USA) version 3.0. (DOC) [file pone.0041261.s001.doc]

Table S1- Primers used in *TP53* sequencing

| Region amplified | Primer sequences (5’🡪3’) | IARC code | Product length |
| --- | --- | --- | --- |
| exon 2-3 | F GATCCCCACTTTTCCTCTTGC | * | 296bp |
|  | R CTCCAGGTCCCCAGCCCAA | * |  |
| exon 4 | F TGCTCTTTTCACCCATCTAC | P-329 | 353bp |
|  | R ATACGGCCAGGCATTGAAGT | P-330 |  |
| exons 5-6 | F TGTTCACTTGTGCCCTGACT | P-236 | 467bp |
|  | R TTAACCCCTCCTCCCAGAGA | P-240 |  |
| exon7 | F CTTGCCACAGGTCTCCCCAA | P-333 | 237bp |
|  | R AGGGGTCAGCGGCAAGCAGA | P-313 |  |
| exon 8-9 | F TTGGGAGTAGATGGAGCCT | P-314 | 445bp |
|  | R AGTGTTAGACTGGAAACTTT | P-315 |  |
| exon 10 | F CAATTGTAACTTGAACCATC | P-E10Li | 260bp |
|  | R GGATGAGAATGGAATCCTAT | P-562 |  |
| exon 11 | F AGACCCTCTCACTCATGTGA | P-E11Le | 245bp |
|  | R TGACGCACACCTATTGCAAG | P-E11Re |  |

Primers previously described at IARC p53 database [23] ([http://www-p53.iarc.fr](http://www-p53.iarc.fr/)). *Designed using Primer Express software (Applied Biosystems, Foster City, CA, USA) version 3.0.
